# Prognostic value of extranodal extension in axillary lymph node-positive breast cancer

**DOI:** 10.1038/s41598-021-88716-4

**Published:** 2021-05-05

**Authors:** XiaoXi Ma, Xia Yang, Wentao Yang, Ruohong Shui

**Affiliations:** 1 Department of Pathology, Fudan University Shanghai Cancer Center, Shanghai, China; 2 Department of Oncology, Shanghai Medical College, Fudan University, Shanghai, China

**Keywords:** Cancer, Medical research, Oncology

## Abstract

Several studies have demonstrated that extranodal extension (ENE) is associated with prognosis in breast cancer. Whether this association should be described in pathological reports warrants further investigation. In this research, we evaluated the predictive value of ENE in axillary lymph nodes (ALNs) in invasive breast cancer and explored the feasibility of employing ENE to predict clinicopathological features, nodal burden, disease recurrence-free survival (DRFS) and overall survival (OS) in clinical practice. In addition, the cutoff values of perpendicular diameter ENE (PD-ENE) and circumferential diameter ENE (CD-ENE) of ENE were investigated. A total of 402 cases of primary invasive breast cancer were extracted from Fudan University Shanghai Cancer Center; these patients underwent axillary lymph node dissection (ALND) between 2010 and 2015. ENE in the ALN was defined as the tumor cells breaking through the lymph node capsule into peripheral adipose tissue and causing connective tissue reactions. Relationships between ENE and clinicopathological features, nodal burden, disease recurrence-free survival (DRFS) and overall survival (OS) were analyzed. PD-ENE was defined by measuring from the point where tumor tissue broke the node capsule to the highest point of the tumor cells in the perinodal adipose tissue.K The average PD-ENE was 1.8 mm; therefore, we divided ENE-positive patients into two groups: PD-ENE no greater than 2 mm and PD-ENE greater than 2 mm. CD-ENE was defined as measuring along the nodal capsule as the distance between peripheral edges of the ENE area. According to the average circumferential diameter (CD-ENE), we classified ENE-positive patients into two groups: CD-ENE no greater than 3 mm and CD-ENE greater than 3 mm. Correlations between ENE cutoffs and prognosis were analyzed. In this cohort of patients, 158 (39.3%) cases were positive for ENE in ALN.98 (24.4%) cases had PD-ENE no larger than 2 mm, and 60 (14.9%) cases had PD-ENE larger than 2 mm. Also, 112 (27.9%) cases had CD-ENE no larger than 3 mm, and 46 (11.4%) cases had CD-ENE larger than 3 mm. Statistical analysis indicated that histological grade, N stage, and HER2 overexpression subtype were associated with ENE. The presence of ENE had significant statistical correlations with nodal burden, including N stage, median metastatic tumor diameter and peri-lymph node vascular invasion (*p* < 0.001, *p* < 0.001, *p* = 0.001, respectively). Cox regression analysis demonstrated that patients with ENE exhibited significantly reduced DRFS in both univariable analysis (HR 2.126, 95% CI 1.453–3.112, *p* < 0.001) and multivariable analysis (HR 1.745, 95% CI 1.152–2.642, *p* = 0.009) compared with patients without ENE. For overall survival (OS), patients with ENE were associated with OS in univariable analysis (HR 2.505, 95% CI 1.337–4.693, *p* = 0.004) but not in multivariable analysis (HR 1.639, 95% CI 0.824–3.260, *p* = 0.159). Kaplan–Meier curves and log-rank test showed that patients with ENE in ALN had lower DRFS and OS (for DRFS: *p* < 0.0001; and for OS: *p* = 0.002, respectively). However, neither the PD-ENE group (divided by 2 mm) nor the CD-ENE group (divided by 3 mm) exhibited significant differences regarding nodal burden and prognosis. Our study indicated that ENE in the ALN was a predictor of prognosis in breast cancer. ENE was an independent prognostic factor for DRFS and was associated with OS**.** ENE in the ALN was associated with a higher nodal burden. The size of ENE, which was classified by a 3-mm (CD-ENE) or 2-mm (PD-ENE) cutoff value, had no significant prognostic value in this study. Based on our findings, the presence of ENE should be included in routine pathological reports of breast cancers. However, the cutoff values of ENE warrant further investigation.

## Introduction

**Figure 1:**
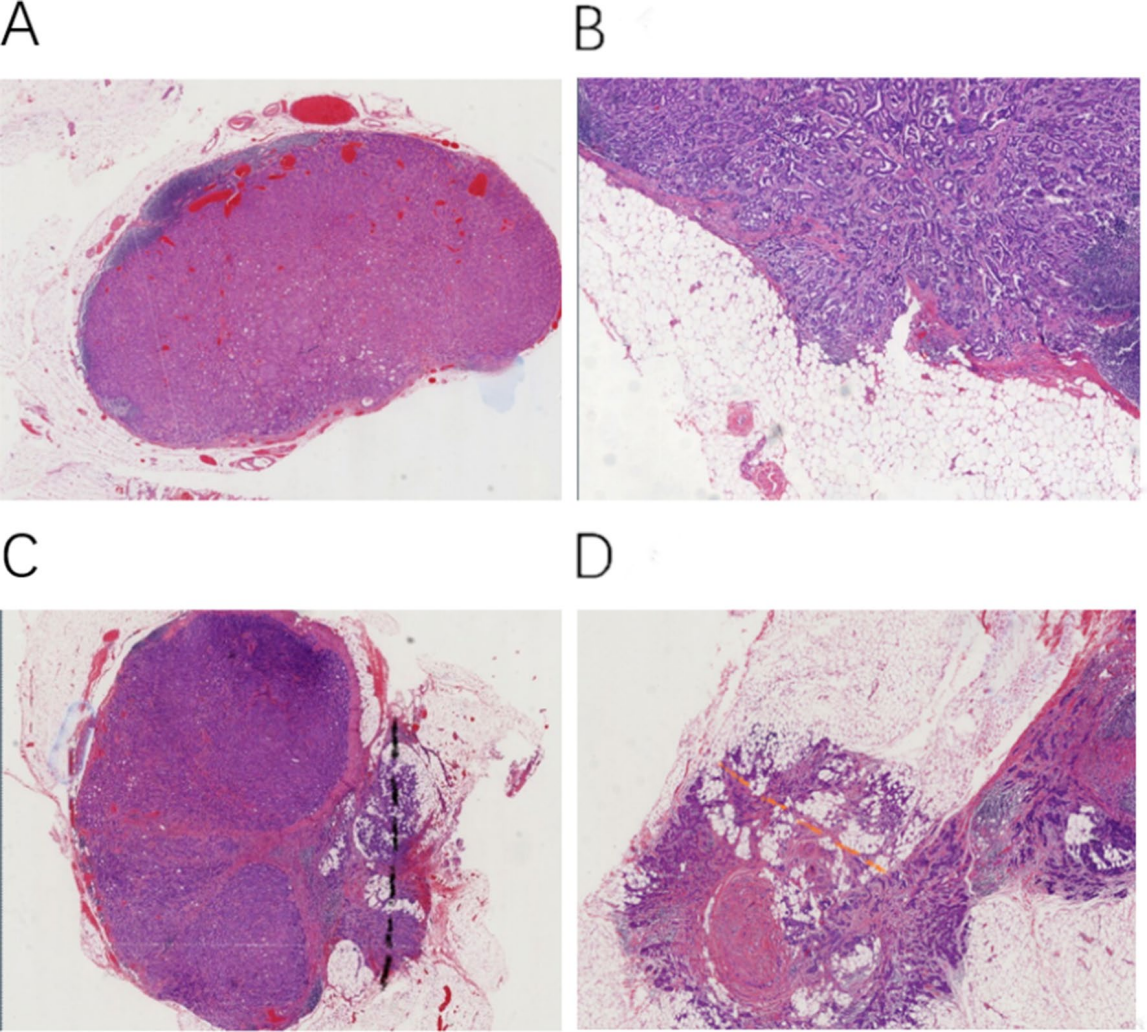
(**A**) Involved axillary lymph node (ALN) without extranodal extension (ENE), (**B**) ALN with ENE, (**C**) the diameter of CD-ENE, (**D**) the diameter of PD-ENE. Original magnification: 200×.

Invasive breast cancer is the most common malignancy in women and has a number of different treatments and prognoses. In 1977, the American Joint Committee on Cancer (AJCC) published the TNM staging system. TNM stage included tumor size (T), nodal status (N), and metastases (M), which were updated consistently. Axillary lymph node metastasis is closely related to the prognosis of breast cancer patients^1,2^. Extranodal extension (ENE) is defined as the tumor cells breaking through the lymph node capsule into peripheral adipose tissue and causing connective tissue reaction (Fig. 1A,B). In 1976, Fisher and his colleagues^3^ reported extranodal extension for the first time, and they believed that ENE in axillary lymph nodes may represent an important prognostic discriminant. In the following decades, many findings have shown that ENE is associated with the number of positive lymph nodes^1, 4,5^ and the prognosis of breast cancer patients^3, 6^.

ENE has been recognized as a prognostic predictor in several types of malignancies^7–12^ and has been included in the AJCC TNM staging system of head and neck cancers^13^. ENE is recommended to be described in routine pathological reports of breast cancers according to the College of American Pathologists (CAP)^14,15^. However, ENE was not included in the eighth edition of the AJCC Cancer staging system of breast cancers^16^, which may be due to the absence of a standardized measurement method and cutoff values for ENE to date.

The study attempted to establish the pathological assessment of ENE in positive axillary lymph nodes and to evaluate the clinical significance of ENE-positive breast cancers, including the association of ENE with clinicopathological parameters, lymph node burden, disease recurrence-free survival (DRFS) and overall survival (OS). In addition, the cutoff value of ENE was explored in this study.

## Materials and methods

### Patients

In this study, 402 patients with primary invasive breast cancer at Fudan University Shanghai Cancer Center from 2010 to 2015 were investigated. All patients underwent axillary lymph node dissection (ALND) with positive axillary lymph nodes and had complete clinical information. Patients with incomplete clinical information, recurrence/metastasis at diagnosis, or previous axillary surgery or who had received neoadjuvant chemotherapy were excluded. Informed consent was obtained from all patients. All tumor tissues and axillary lymph nodes were fixed in 10% neutral formalin, embedded in paraffin wax and examined using hematoxylin and eosin (H&E) staining. Each lymph node was sliced with the largest profile. According to National Comprehensive Cancer Network (NCCN) guideline recommendations and patients’ intention, all patients were treated with surgery (breast conserving resection or mastectomy with ALN dissection) with or without radiotherapy, systematic chemotherapy, and endocrine therapy. Among this cohort of patients, 391 (97.1%) received chemotherapy, 333 (82.7%) received radiotherapy, 301 (74.6%) received endocrine therapy and 69 (17.2%) received targeted therapy.

### Patient characteristics

**Figure 2:**
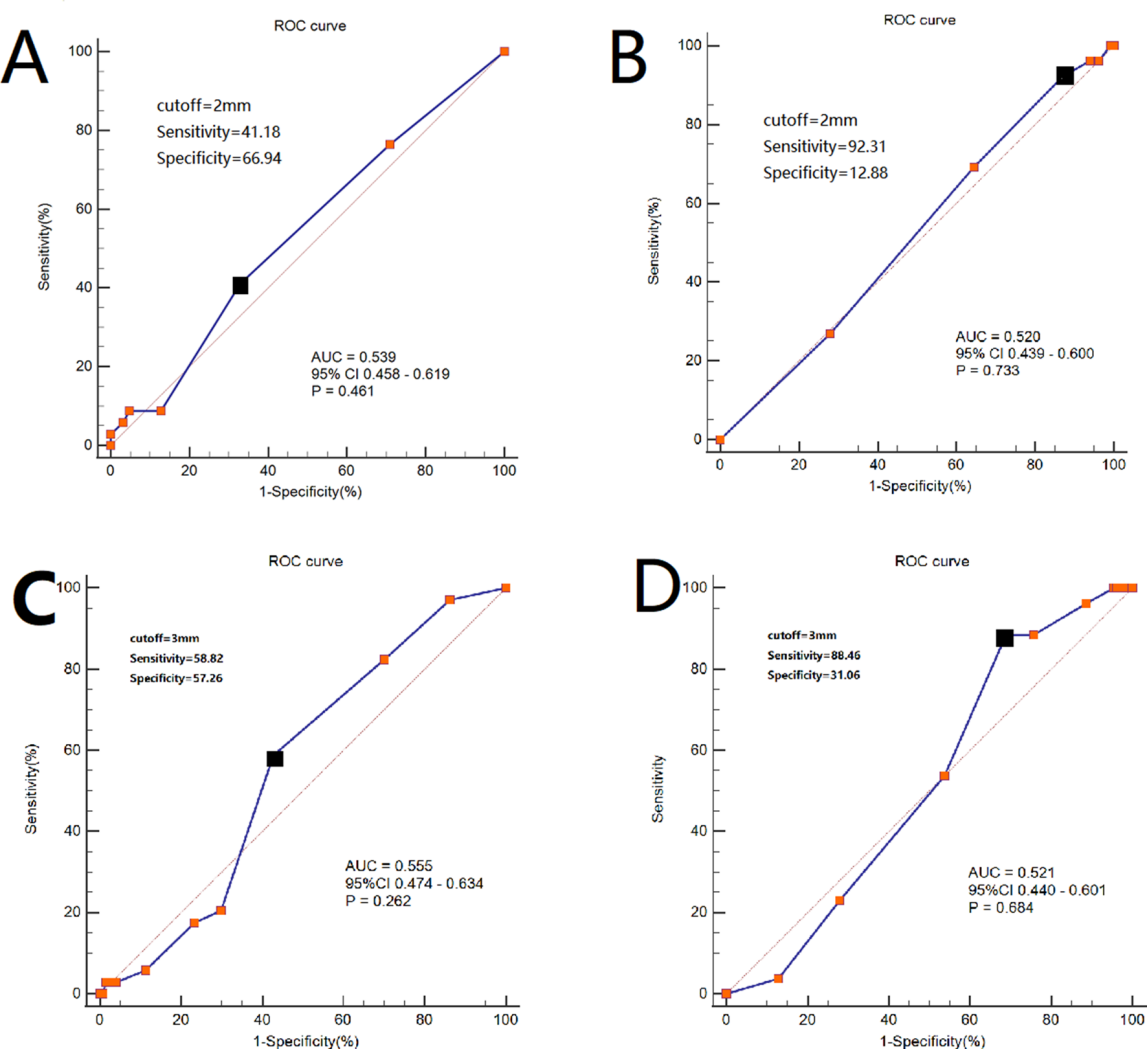
Receiver operating characteristics (ROC) analysis for the cutoffs of ENE for DFS and OS. (**A**) ROC curve of PD-ENE to predict DFS. (**B**) ROC curve of PD-ENE to predict OS. (**C**) ROC curve of CD-ENE to predict DFS. (**D**) ROC curve of CD-ENE to predict OS. The black dot indicated the optimal threshold. The area under the curve (AUC), 95% confidence interval (CI) and *p*-value were listed in the picture.

Two senior breast pathologists reviewed clinicopathological features. The presence and size of ENE, median metastatic tumor diameter, and peri-lymph node vascular invasion were reviewed by two breast pathologists in a blinded way. The clinicopathological features included patient age, histological grade, T stage, N stage, estrogen receptor (ER) status, progesterone receptor (PR) status, human epidermal growth factor receptor 2 (HER2) status, and peri-lymph node vascular invasion. Nodal burden included N stage, median metastatic tumor diameter, number of axillary lymph nodes, peri-lymph node vascular invasion and ENE foci. Molecular subtype, disease recurrence-free survival (DRFS) and overall survival (OS) were also analyzed. ER and PR were judged as positive if ≥ 1% of tumor cells showed nuclear staining in immunohistochemistry (IHC)^17^. HER2 was judged as positive by HER2 protein IHC 3 + score or HER2 gene amplification by fluorescent in situ hybridization (FISH) detection^18^. Metastatic tumor diameter was defined as the maximum diameter of tumor metastasis in positive lymph nodes. Peri-lymph node vascular invasion was defined as the presence of tumor cells in the vessels surrounding the lymph nodes. The molecular subtypes included the luminal-A-like subtype, luminal-B-like subtype, HER2-overexpression subtype and triple negative breast cancer (TNBC)^19–21^. The presence and size of ENE in ALN was evaluated. ENE in the ALN was defined as tumor tissue breaking through the nodal capsule into peripheral adipose tissue with or without an associated desmoplastic stromal response (i.e., inflamed granulation tissue and/or fibrosis). ENE size was measured as the highest (perpendicular diameter ENE, PD-ENE) or widest (circumferential diameter ENE, CD-ENE) diameter of the invasive front of ENE. PD-ENE was defined as measuring from the point where the tumor tissue breaks the node capsule to the highest point of the tumor cells in the perinodal adipose tissue (Fig. 1D). CD-ENE was defined as measuring along the nodal capsule to determine the distance between peripheral edges of the ENE area (Fig. 1C). The original data of PD-ENE and CD-ENE both followed normal distribution, so we observed the average and median values of the data. The average and median of PD-ENE were 1.8 mm and 2 mm, respectively, and the average and median of CD-ENE were 2.9 mm and 3 mm, respectively. Then we did a sensitivity analysis related to the cut-off values, receiver operating characteristics (ROC) curve analysis was used to identify the cut-off values for PD-ENE and CD-ENE to predict DRFS and OS. Cutoff value of 2 mm for PD-ENE had relatively higher sensitivity and specificity to predict DRFS and OS, compared with 1 mm and 3 mm (Fig. 2, Table 1). It was revealed that the area under the curve (AUC) of PD-ENE level was 0.539 (95% CI 0.458–0.619, *P* = 0.461) and the relatively optimal cutoff value of PD-ENE to predict DRFS was 2 mm (Fig. 2A), the sensitivity and specificity are 41.18% and 66.94% (Table 1). The curve (AUC) of PD-ENE level was 0.520 (95% CI 0.439–0.600, *p* = 0.733) and the relatively optimal cutoff value of PD-ENE to predict OS was 2 mm (Fig. 2B), the sensitivity and specificity are 92.31% and 12.88% (Table 1). Cutoff value of 3 mm for CD-ENE had relatively higher sensitivity and specificity to predict DRFS and OS, compared with 1 mm, 2 mm and 4 mm (Fig. 2, Table 1). It was revealed that the area under the curve (AUC) of CD-ENE level was 0.555 (95% CI 0.474–0.834, *P* = 0.461) and the relatively optimal cutoff value of CD-ENE to predict DRFS was 3 mm (Fig. 2C), the sensitivity and specificity are 58.82% and 57.26% (Table 1). The curve (AUC) of CD-ENE level was 0.521 (95% CI 0.440–0.601, *P* = 0.684) and the relatively optimal cutoff value of CD-ENE to predict OS was 3 mm (Fig. 2D), the sensitivity and specificity are 88.46% and 31.06% (Table 1). So we divided subgroups by 2-mm (PD-ENE) and 3-mm (CD-ENE) cutoffs.

**Table 1:** Comparisons between different cut-off values of PD-ENE.

Cut-off values	No. of patients (%)	Sensitivity (%)	Specificity (%)	Youden’s index
**PD-ENE for DFS**				
1 mm	37.3	76.47	29.03	0.055
2 mm	22.7	41.18	66.94	*0.081*
3 mm	6.3	8.82	87.1	− 0.041
**PD-ENE for OS**				
1 mm	37.3	69.23	35.61	0.048
2 mm	22.7	92.31	12.88	*0.052*
3 mm	6.3	96.15	6.06	0.022
**CD-ENE for DFS**				
1 mm	15.8	97.06	13.71	0.107
2 mm	26.5	82.35	29.84	0.122
3 mm	18.3	58.82	57.26	*0.161*
4 mm	5.6	20.59	70.16	− 0.093
**CD-ENE for OS**				
1 mm	15.8	23.08	71.97	− 0.050
2 mm	26.5	53.85	46.21	0.060
3 mm	18.3	88.46	31.06	*0.195*
4 mm	5.6	88.46	24.24	0.127

PD-ENE perpendicular diameter of extra-nodal extension; CD-ENE circumferential diameter of extra-nodal extension; DFS disease recurrence free survival; OS overall survival; Youden’s Index = sensitivity + specificity − 1. The values in italic are the maximum Youden's index.

### Study end points

This study primarily investigated the relationships between ENE in ALN and clinicopathological features, nodal burden, molecular subtype, DRFS and OS. After undergoing surgery for primary breast cancer, patients were assessed for disease recurrence or/and metastasis by following standard clinical practice. DRFS was defined as the time from surgery to events including local recurrence, distant recurrence, or death resulting from any cause (whichever occurred first). OS was defined as the time from surgery to death from any cause.

### Statistical analysis

All statistical analyses were carried out using IBM SPSS Statistics 21.0. All figures were depicted using GraphPad Prism7 (GraphPad Software). The χ^2^ test or Fisher’s exact test was used to test categorical variables. The distributional assumption was checked and the data were approximately normally distributed, so variables were analyzed in different ENE groups using independent t-test. Logistic regression analysis was used to evaluate relationships between clinicopathological parameters and ENE in a univariable model and in a multivariate model. The variables had a statistical relationship with ENE (*p* ≤ 0.05) in univariable analysis were chosen for multivariable analysis. Before performing Cox regression analysis, the Proportional Hazards assumption (PH assumption) was checked and it was satisfied. Cox regression analysis was used to analyze the correlations between ENE and DRFS or OS in a univariable model and a multivariable model. The variables had a statistical relationship with prognosis (*p* ≤ 0.05) in univariable analysis were chosen for multivariable analysis. The Kaplan–Meier method and log-rank test were used to analyze the relationship between ENE in ALN and the duration of DRFS or OS. Two-sided exact tests were employed, and *p*-values < 0.05 were considered to be significant.

### Ethics approval and consent to participate

The Ethics Institutional Review Board of Fudan University Shanghai Cancer Center approved this study. According to the ethics standards of the Ethics Institutional Review Board of Fudan University Shanghai Cancer Center and with the 1964 Helsinki Declaration and its later amendments, all human-related procedures met the standards. All patients who participated in the study signed informed consent forms, allowing us to use their organizational materials, conduct scientific projects and release data.

## Results

### ENE in ALN and clinicopathological features

All breast cancer patients who entered the inclusion criteria are listed in Table 2. This cohort of 402 female patients all underwent axillary lymph node dissection (ALND). The median age of all patients was 52 years (ranging from 30 to 83 years). A total of 158/402 (39.3%) patients had positive ENE in the ALN. Statistical analysis showed that patients with ENE in ALN were associated with histological grade (*p* = 0.022), N stage (*p* < 0.001), and peri-lymph node vascular invasion (*p* = 0.001) compared with patients without ENE in ALN. However, there were no significant differences between ENE and patient median age, T stage, ER status, or PR status HER-2 status (Table 2).

**Table 2:** Correlations between ENE in ALN and clinicopathological parameters.

Variables		No. of patients (%)	ENE		Univariable analysis			Multivariable analysis		
			Negative	Positive	OR	95% CI	*p*-value	OR	95% CI	*p*-value
Total population		402 (100)	244 (60.7%)	158 (39.3%)						
Median age (Y)		52 (30–83)	50 (30–80)	54 (32–83)						
T stage							0.465			
	T1	119 (29.6)	70 (17.4)	49 (12.2)	1	–	–			
	T2	261 (64.9)	162 (40.3)	99 (24.6)	0.873	0.561–1.359	0.547			
	T3	22 (5.5)	12 (3.0)	10 (2.5)	1.190	0.477–2.973	0.709			
Histological grade	2	207 (51.5)	115(28.6)	92 (22.9)	1	–	–			
	3	195 (48.5)	130 (32.3)	65 (16.2)	1.632	1.089–2.445	0.018	0.559	0.347–0.889	0.016
N stage							< 0.001			< 0.001
	1	107 (26.6)	87 (21.6)	20 (5.0)	1	–	–			
	2	191 (47.5)	119 (29.6)	72 (17.9)	2.574	1.459–4.451	0.001	2.456	1.360–4.434	0.003
	3	104 (25.9)	38 (9.5)	66 (16.4)	7.877	4.194–14.796	< 0.001	7.301	3.618–14.735	< 0.001
ER status	Negative	96 (23.9)	63 (15.7)	33 (8.2)	1	–	–			
	Positive	306 (76.1)	181 (45.0)	125 (31.1)	0.758	0.470–1.224	0.258			
PR status	Negative	121 (30.1)	78 (19.5)	43 (10.6)	1	–	–			
	Positive	281 (69.9)	166 (41.3)	115 (28.6)	0.796	0.512–1.238	0.331			
HER2 status	Negative	307 (76.4)	180 (44.8)	127 (31.6)	1	–	–			
	Positive	95 (23.6)	64 (15.9)	31 (7.7)	1.457	0.897–2.367	0.129			
Lympho-vascular invasion	Negative	206 (51.2)	142 (35.3)	64 (15.9)	1	–	–			
	Positive	196 (48.8)	102 (25.4)	94 (23.4)	2.045	1.361–3.072	0.001	1.258	0.793–1.994	0.330
Molecular subtype	Luminal-A like	72 (17.9)	37 (9.2)	35 (8.7)	1	–	–			
	Luminal-B like	182 (45.3)	114 (28.4)	68 (16.9)	0.631	0.363–1.094	0.101	0.661	0.361–1.211	0.180
	HER2-overexpression	52 (12.9)	36 (9.0)	16 (3.9)	0.470	0.222–0.993	0.048	0.418	0.179–0.977	0.044
	Triple negative breast cancer	45 (11.2)	31 (7.7)	14 (3.5)	0.477	0.218–1.044	0.064	0.566	0.232–1.381	0.211
	Others	51 (12.7)	/	/						

ER estrogen receptor; PR progesterone receptor; HER2 human epidermal growth factor receptor 2; ENE extranodal extension.

In this cohort of patients, 72 (17.9%) cases were luminal-A-like subtype, 182 (45.3%) were luminal-B-like subtype, 52 (12.9%) were HER2-overexpression subtype and 45 (11.2%) were TNBC. Logistic regression indicated that the HER2-overexpression subtype (*p* = 0.048) was associated with the presence of ENE in univariable analysis. Multivariable analysis demonstrated that the HER2 overexpression subtype (OR 0.418, 95% CI 0.179–0.977, *p* = 0.044) was an independent predictor of ENE. However, the remaining three molecular typing and ENE were not significant (Table 2).

### ENE in ALN and nodal burden

Compared with patients without ENE in ALN, patients with ENE were associated with nodal burden. The presence of ENE had significant statistical correlations with N stage, median metastatic tumor diameter and peri-lymph node vascular invasion (*p* < 0.001, *p* = 0.001, *p* = 0.001, respectively), while the number of removed axillary lymph nodes had no significant correlations with ENE (*p* = 0.111). The median metastatic tumor diameter was 0.7 cm (range 0.1–2.8). The average diameter of PD-ENE was 1.8 mm. The average diameter of CD-ENE was 2.9 mm. There were no significant differences among the PD-ENE groups in nodal burden, but differences were also not observed among the CD-ENE groups. In addition, the two PD-ENE groups had no statistical consistency in the number of ENE foci, nor did the CD-ENE groups (Table 3).

**Table 3:** ENE in ALN and nodal burden.

Nodal burden	ENE					*p*-value		
	Negative (244)	Positive (158)				Negative vs Positive	≤ 2 mm vs > 2 mm	≤ 3 mm vs > 3 mm
		PD-ENE		CD-ENE				
		≤ 2 mm (98)	> 2 mm (60)	≤ 3 mm (112)	> 3 mm (46)			
**N stage**								
N1	87	14	6	13	7			
N2	120	43	28	50	21			
N3	37	41	26	49	18	< 0.001	0.843	0.779
**No. of removed axillary lymph nodes**								
≤ 20	154	61	26	63	24			
> 20	90	37	34	49	22	0.111	0.427	0.645
**Median metastatic tumor diameter (cm)**								
≤ 0.7	120	29	21	36	14			
> 0.7	124	69	39	76	32	< 0.001	0.497	0.835
**Peri lymph node vascular invasion**								
No	142	40	24	46	18			
Yes	102	58	36	66	28	0.001	0.748	0.823
**No. of ENE foci**								
1–2		71	45	82	34			
> 2		27	15	30	12		0.283	0.929

ENE extranodal extension; PD-ENE perpendicular diameter of extra-nodal extension; CD-ENE, circumferential diameter of extra-nodal extension.

### ENE in ALN and prognosis

The median follow-up month of patients was 69 months (range 1–117). In the 158 patients with ENE in ALN, 63 (39.8%) had distant metastasis compared with 46/244 (18.8%) patients without ENE in ALN (*p* < 0.001). In this cohort, survival data were available for all patients. Cox’s proportional hazards method showed that ENE in ALN was associated with DRFS (HR 2.126, 95% CI 1.453–3.112, *p* < 0.001) and OS (HR 2.505, 95% CI 1.337–4.693, *p* = 0.004) in univariable analysis. Multivariable analysis showed that ENE in ALN was an independent predictor of DRFS (HR 1.745, 95% CI 1.152–2.642, *p* = 0.009), while no statistical significance was shown for OS (HR 1.639, 95% CI 0.824–3.260, *p* = 0.159) (Table 4).

**Table 4:** Correlations between ENE in ALN and DRFS and OS.

Variables	Disease recurrence free survival				Overall survival			
	Univariable analysis		Multivariable analysis		Univariable analysis		Multivariable analysis	
	HR (95% CI)	*p*-value	HR (95% CI)	*p*-value	HR (95% CI)	*p*-value	HR (95% CI)	*p*-value
**Median age (Y)**								
≤ 52								
> 52	0.715 (0.491–1.041)	0.080			0.660 (0.480–0.908)	0.011	2.081 (1.0814.006)	0.028
**T stage**		0.385				0.004		
T1	–	–			–	–		
T2	1.526 (0.970–2.402)	0.067			3.213 (1.254–8.230)	0.015	2.932 (1.122–7.661)	0.028
T3	1.920 (0.863–4.273)	0.110			3.416 (0.814–14.330)	0.093	2.352 (0.542–10.218)	0.254
**Histologic grade**								
2								
3	0.914 (0.753–1.110)	0.364			0.777 (0.568–1.062)	0.114		
**N stage**		< 0.001				< 0.001		
1	–	–			–	–		
2	1.367 (0.776–2.411)	0.279	1.201 (0.671–2.148)	0.537	1.893 (0.610–5.875)	0.269	1.702 (0.534–5.424)	0.369
3	3.716 (2.180–6.336)	< 0.001	2.781 (1.501–5.154)	0.001	7.683 (2.673–22.086)	< 0.001	4.677 (1.440–15.192)	0.010
**No. of removed axillary lymph nodes**								
≤ 20								
> 20	0.920 (0.762–1.112)	0.389						
**Median metastatic tumor diameter (cm)**								
≤ 0.7								
> 0.7	1.817 (1.212–2.725)	0.004	1.186 (0.760–1.851)	0.452	1.946 (0.990–3.825)	0.053	0.896 (0.428–1.875)	0.771
**ENE**								
Negative								
Positive	2.126 (1.453–3.112)	< 0.001	1.745 (1.152–2.642)	0.009	2.505 (1.337–4.693)	0.004	1.639 (0.824–3.260)	0.159
**ER status**								
Negative								
Positive	1.130 (0.910–1.403)	0.267			1.502 (1.097–2.058)	0.011	0.908 (0.297–2.779)	0.866
**PR status**								
Negative								
Positive	1.214 (0.987–1.492)	0.066	0.858 (0.540–1.364)	0.519	1.463 (1.071–1.999)	0.017	0.678 (0.232–1.978)	0.477
**HER2 status**								
Negative								
Positive	0.758 (0.613–0.937)	0.010	1.743 (1.085–2.799)	0.022	0.680 (0.494–0.938)	0.019	1.500 (0.708–3.180)	0.290
**Peri lymph node vascular invasion**								
No								
Yes	1.405 (0.958–2.060)	0.082	0.852 (0.559–1.298)	0.456	2.020 (1.059–3.855)	0.033	1.169 (0.571–2.392)	0.670

ER estrogen receptor; PR progesterone receptor; HER2 human epidermal growth factor receptor 2; ENE extranodal extension.

**Figure 3:**
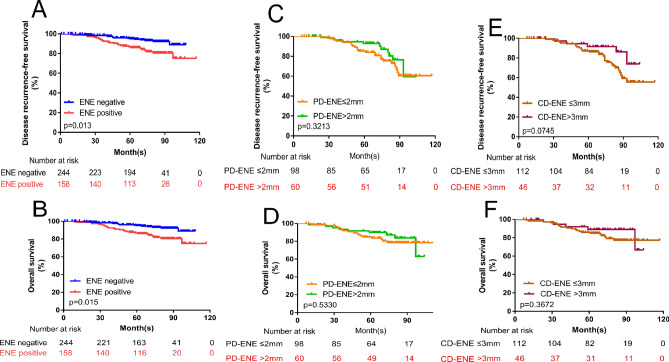
Kaplan–Meier curves and Log-rank test show associations of ENE in ALN with DRFS and OS. (**A**,**B**) Comparison of survival rate for DRFS (ENE negative group vs. ENE positive group: *p* < 0.0001) (**A**) and OS (ENE negative group vs. ENE positive group: *p* = 0.002) (**B**) Between different ENE groups in whole population. (**C**–**F**) Comparison of survival rate for DRFS (PD-ENE no larger than 2 mm group vs. PD-ENE larger than 2 mm group: *p* = 0.632) (**C**) and OS (PD-ENE no larger than 2 mm group vs. PD-ENE larger than 2 mm group: *p* = 0.345) (**D**) Comparison of survival rate for DRFS (CD-ENE no larger than 3 mm group vs. CD-ENE larger than 3 mm group: *p* = 0.581) (**E**) and OS (CD-ENE no larger than 3 mm group vs. CD-ENE larger than 3 mm group: *p* = 0.880) (**F**) between different ENE groups in patients with ENE in ALN.

**Figure 4:**
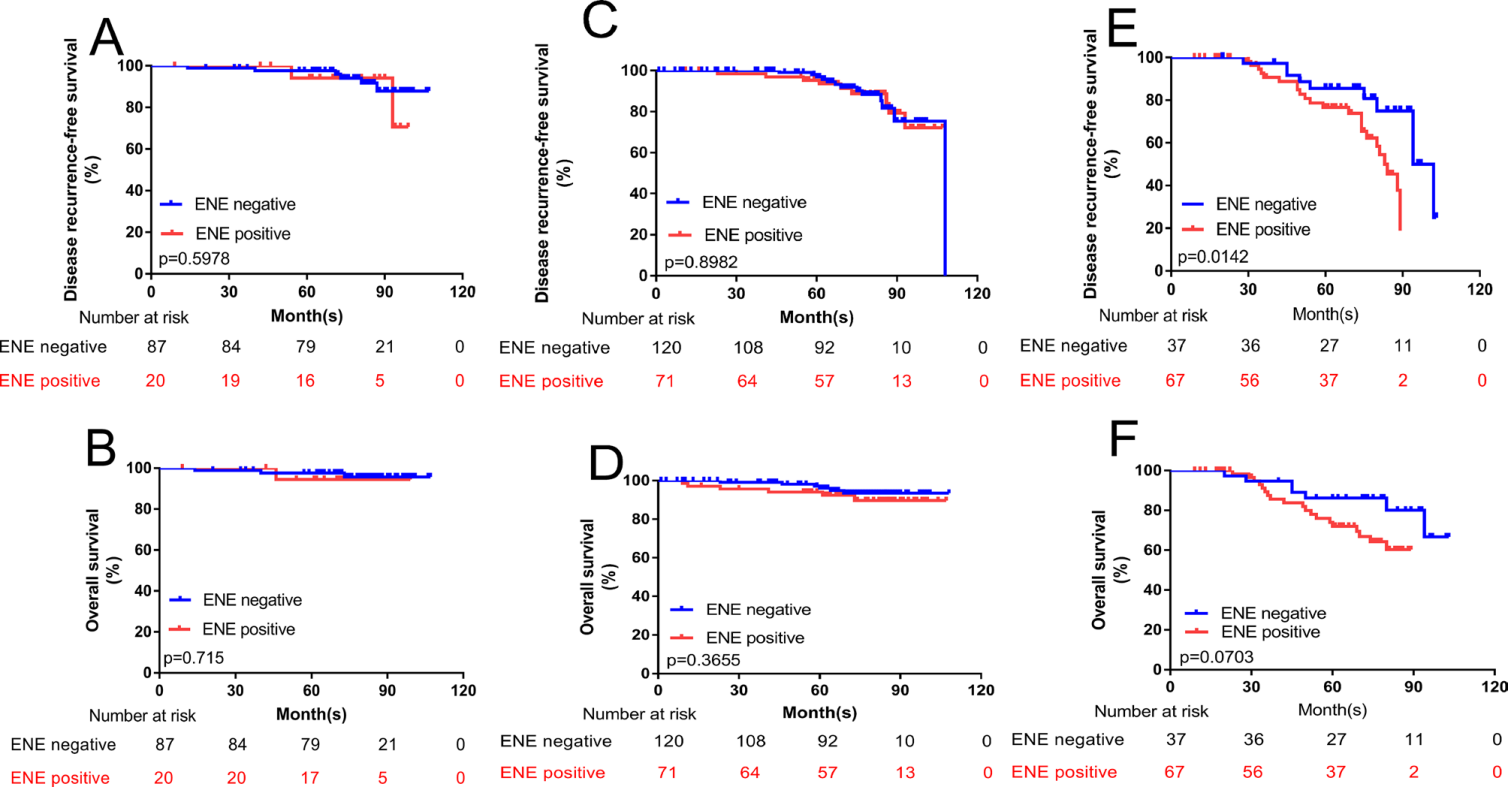
Kaplan–Meier curves and Log-rank test show associations of ENE in ALN with DRFS and OS in whole patients with different nodal (N) stage. (**A**,**B**) Comparison of survival rate for DRFS (ENE negative group vs. ENE positive group: *p* = 0.557) (**A**) and OS (ENE negative group vs. ENE positive group: *p* = 0.547) (**B**) between different ENE groups in patients with N1 stage. (**C**,**D**) Comparison of survival rate for DRFS (ENE negative group vs. ENE positive group: *p* = 0.919) (**C**) and OS (ENE negative group vs. ENE positive group: *p* = 0.367) (**D**) between different ENE groups in patients with N2 stage. (**E**–**F**) Comparison of survival rate for DRFS (ENE negative group vs. ENE positive group: *p* = 0.047) (**E**) and OS (ENE negative group vs. ENE positive group: *p* = 0.346) (**F**) between different ENE groups in patients with N3 stage.

The DRFS and OS of patients with SLN involvement were classified according to ENE (Fig. 3A,B), N1 stage (Fig. 4A,B), N2 stage (Fig. 4C,D) and N3 stage (Fig. 4E,F). The DRFS and OS in those patients with ENE in ALN were categorized by PD-ENE (Fig. 3C,D) and CD-ENE (Fig. 3E,F). Kaplan–Meier curves and log-rank tests showed that patients with ENE in the ALN group had poorer outcomes than did those in the ENE-negative group (for DRFS: *p* < 0.001; and for OS: *p* = 0.002, respectively). Patients in the N3 stage who had ENE in the ALN had significantly lower DRFS but not OS.

In ENE-positive patients, Cox multivariable regression analysis indicated that the number of ENE foci and median metastatic tumor diameter were independent factors for DRFS, and the number of ENE foci was also an independent prognostic factor of OS. However, the size of ENE (PD-ENE and CD-ENE) subdivided by 2 mm (or 3 mm) cutoff values was not an independent factor for DRFS and OS in these patients (Table 5). Kaplan–Meier curves and log-rank tests showed that the size of ENE (PD-ENE and CD-ENE) subdivided by 2 mm (or 3 mm) cutoff values was not significant in DRFS and OS (Fig. 3C,D).

**Table 5:** Correlations between ENE cutoffs and prognosis.

Variables	Disease recurrence free survival				Overall survival			
	Univariable analysis		Multivariable analysis		Univariable analysis		Multivariable analysis	
	HR (95% CI)	*p*-value	HR (95% CI)	*p*-value	HR (95% CI)	*p*-value	HR (95% CI)	*p*-value
**Median age (Y)**								
≤ 52								
> 52	0.645 (0.390–1.066)	0.087			0.532 (0.235–1.205)	0.132		
**T stage**		0.794				0.346		
T1	–	–			–	–		
T2	1.533 (0.867–2.712)	0.142			3.543 (1.052–11.926)	0.041	3.324 (0.939–11.769)	0.063
T3	1.075 (0.311–3.719)	0.909			3.210 (0.534–19.287)	0.202	2.486 (0.411–15.027)	0.321
**Histologic grade**								
2								
3	0.773 (0.497–0.999)	0.049	1.072 (0.606–1.894)	0.812	0.686 (0.461–1.022)	0.064		
**N stage**		0.002				0.003		
1	–	–			–	–		
2	0.694 (0.269–1.793)	0.451			1.726 (0.208–14.339)	0.614		
3	2.280 (0.966–5.382)	0.060			6.051 (0.807–45.370)	0.080		
**No. of removed axillary lymph nodes**								
≤ 20								
> 20	0.829 (0.646–1.065)	0.142			0.871 (0.588–1.290)	0.492		
**Median metastatic tumor diameter (cm)**								
≤ 0.7								
> 0.7	2.183 (1.190–4.006	0.012	1.907 (1.007–3.610)	0.047	1.642 (0.654–4.124)	0.291		
**No. of ENE foci**								
1–2								
> 2	2.050 (1.224–3.434)	0.006	2.080 (1.220–3.545)	0.007	2.743 (1.244–6.049)	0.012	0.328 (0.143–0.751)	0.008
**CD-ENE**								
≤ 3 mm								
> 3 mm	1.138 (0.668–1.938)	0.634			1.179 (0.514–2.706)	0.697		
**PD-ENE**								
≤ 2 mm								
> 2 mm	1.144 (0.695–1.883)	0.598			0.714 (0.321–1.591)	0.410		
**ER status**								
Negative								
Positive	1.732 (1.042–1.806)	0.024	1.190 (0.483–2.930)	0.706	1.709 (1.143–2.556)	0.009	1.831 (0.479–7.003)	0.377
**PR status**								
Negative								
Positive	1.547 (1.174–2.039)	0.002	0.487 (0.207–1.147)	0.100	1.774 (1.189–2.649)	0.005	2.391 (0.687–8.328)	0.171
HER2 status								
Negative								
Positive	0.690 (0.512–0.931)	0.015	1.736 (0.859–3.507)	0.125	0.640 (0.419–0.979)	0.040	0.934 (0.334–2.612)	0.897
**Peri lymph node vascular invasion**								
No								
Yes	1.067 (0.634–1.796)	0.808			1.047 (0.470–2.335)	0.910		

ENE extranodal extension; PD-ENE perpendicular diameter of extra-nodal extension; CD-ENE, circumferential diameter of extra-nodal extension.ER estrogen receptor; PR progesterone receptor; HER2 human epidermal growth factor receptor 2.

## Discussion

Breast cancer is a heterogeneous disease and has poor prognosis. To help the clinician analyze the patient's condition, choose the treatment plan and judge the prognosis, the AJCC proposed the TNM staging system. This system considers tumor size, nodal status and metastasis. As physicians deepen their understanding of breast tumors, an increasing number of criteria have been added to this evaluation system, including immunohistochemistry and biomarkers. ENE was defined as the tumor cells breaking through the lymph node capsule into peripheral adipose tissue and causing connective tissue reactions. ENE was included in the N staging criteria for oral squamous cell carcinoma in the eighth edition of the AJCC^13^, but was not included in the staging criteria for breast cancer^16^. CAP mentioned that ENE should be included in routine pathology reports^22^. Therefore, we explored the relationship between ENE and clinicopathological parameters, nodal burden or prognosis to determine whether ENE should be listed in our standardized pathology report for breast cancer, and if it needs to be included, the ENE cutoff values need to be identified.

In this retrospective analysis that included 402 invasive breast cancers with ALN involvement, 158 patients (39.3%) were ENE-positive in ALN, which is fewer than were observed by Palamba et al. (63.9%)^4^ and Aziz et al. (53.2%)^6^. The presence of ENE was associated with clinicopathological parameters, including histological grade and molecular subtype. However, the relationships between ENE and histological grade were not mentioned in recently published studies. The statistical analysis of the correlations between the molecular subtype of breast cancer and ENE showed that the HER2 overexpression subtype was an independent predictor of the presence of ENE, which has not been widely observed in recent studies. Ahmed, ARH and his colleagues^23^ showed that HER-2 expression in pT1 and pT2 tumors elevated the risk of ALN metastasis by 7.7-fold and 7.6-fold, showed that HER-2 status expression is a strong independent predictor of nodal metastasis in breast cancer. Statistical analysis demonstrated respectively, and grade 1 and 2 tumors that expressed HER2 were 16.0 and 7.8 times more likely to have ALN metastasis, respectively.

In our research, ENE-positive patients had significant differences in nodal burden, including N stage, median metastatic tumor diameter, and peri-lymph node vascular invasion, compared with ENE-negative patients. This result is in keeping with the findings of several studies that indicated that ENE has significant relationships with nodal burdens^1,2,4,5,24^. Palamba et al. and Abdessalam et al. demonstrated that the presence of extranodal extension in axillary lymph node metastases was a good predictor for the number of positive nodes^1,4^. Ahmad et al. demonstrated that there were significant associations between the number of positive nodes and perinodal extension^25^. However, the median metastatic tumor diameter and perinodal vascular invasion were not mentioned in the current literature. Cox proportional hazards regression analyses indicated that the presence of ENE was an independent predictor of DRFS (HR 1.745, 95% CI 1.152–2.642, *p* = 0.009) but not OS (HR 1.639, 95% CI 0.824–3.260, *p* = 0.159). This result was in keeping with the findings of Dobi and his colleagues, who showed that extracapsular tumor spread (ECS) status was an independent factor for DRFS (HR 0.7, 95% CI 0.49–0.96, *p* = 0.03) but not OS^26^. Although this result was similar to the results of this study, Dobi et al. only analyzed early breast cancer, and our research included all staging patients. Bucci et al. demonstrated that the presence of extranodal spread (ENS) was significantly associated with poor DRFS (*p* = 0.013)^27^. Nottegar et al. demonstrated that ENE was associated with a higher risk of both mortality and recurrence of disease^28^. Some studies focused on ENE in early breast cancer patients^26,29,30^. Kanyilmaz et al.^30^ demonstrated that the extent of extracapsular extension was an important prognostic factor for survival in pT1-2N1 breast cancer patients. In our study, statistical analysis demonstrated that ENE in N3-stage patients was significantly correlated with prognosis, while there was no significant relationship between ENE and prognosis in N1- and N2-stage patients.

The cutoff value of ENE has been investigated in the literature. Aziz et al. divided the clinical significance of ENE into circumferential (CD-ENE) and perpendicular (PD-ENE) extranodal growth, and the results showed that PD-ENE (with 3 mm serving as the cutoff value) was an independent prognostic factor for disease-free survival of breast cancers, while CD-ENE was not associated with prognosis^6^. Palamba et al. subdivided extranodal extension-positive patients into a minimal extranodal extension (MEE) group and an extensive extranodal extension (EEE) group. The EEE group had a greater number of positive nodes than did the MEE group (*p* < 0.001), but the prognostic value of this discrepancy was not explored^4^. Kanyilmaz et al. divided ENE into five grades: grade 0: tumor within the side of the lymph node or tumor within the nodal capsular sinus with no thickening of the lymph node capsule; grade 1: tumor encompassing the subcapsular sinus with thickening of the lymph node capsule; grade 2: tumor spreading ≤ 1 mm beyond the lymph node capsule; grade 3: tumor spreading > 1 mm beyond the lymph node capsule; grade 4: no residual lymph node tissue. This research showed that the presence of ECE was an independent predictor for survival outcomes in pT1-2N1 breast cancer patients, and grade 3–4 ECE appeared to be associated with a lower OS and DRFS^30^. However, Kanyilmaz only explored the prognostic value of ECE on the prognosis of patients in N1 stage. In our study, ENE was classified into CD-ENE and PD-ENE by 3-mm and 2-mm cutoffs, respectively. However, Cox proportional hazards regression analyses indicated that neither CD-ENE (with 3 mm serving as the cutoff value) nor PD-ENE (with 2 mm serving as the cutoff value) had a significant relationship with DRFS or OS, which demonstrated that the presence of ENE in ALN, either subdivided by a 2-mm cutoff value or 3-mm cutoff value, had no predictive value in invasive breast cancer.

Our study had several limitations. First, it was a single-center retrospective analysis and included a smaller sample size. We need to perform multicenter studies and large-scale prospective and retrospective studies to investigate the prognostic value of ENE in invasive breast cancer. Meanwhile, the cutoff values of ENE warrant further investigation.

## Conclusion

Our study indicated that ENE in ALN was a predictor for prognosis in breast cancer. ENE was an independent prognostic factor for DRFS and was associated with OS**.** ENE in the ALN was associated with a higher nodal burden. The size of ENE, which was classified by a 3-mm (CD-ENE) or 2-mm (PD-ENE) cutoff value, had no significant prognostic value in this study. Based on our findings, the presence of ENE should be included in routine pathological reports of breast cancers. However, the cutoff values of ENE warrant further investigation.
